# Advancing GIS-based suitability analysis of BtX, PtX, PBtX, and eBtX facilities using the fuzzy analytic hierarchy process

**DOI:** 10.1016/j.mex.2025.103194

**Published:** 2025-01-29

**Authors:** Marcel Dossow, Mengxi Chen, Hartmut Spliethoff, Sebastian Fendt

**Affiliations:** Technical University of Munich, Chair of Energy Systems, Boltzmannstr. 15, 85748, Garching b. München, Germany

**Keywords:** Biomass-to-X, Power-and-biomass-to-X, Power-to-X, Electrified biomass-to-X, Fuzzy analytic hierarchy process, Geographic information systems, Multi-criteria decision analysis, Renewable electricity, TUM Chair of Energy Systems GIS-based Suitability Analysis using the Fuzzy Analytic Hierarchy Process for optimal BtX, PtX, PBtX, and eBtX plant siting (CES-GIS-SAFAHP)

## Abstract

To address the urgent need for sustainable fuel production, this study proposes a novel methodology that integrates Geographic Information Systems (GIS) and Multi-Criteria Decision Analysis (MCDA) techniques to identify optimal sites for Biomass-to-X (BtX), Power-to-X (PtX), or hybrid (e-/PBtX) facilities. The proposed methodology provides a systematic and quantitative approach to evaluate location suitability, offering valuable insights for spatial decision-making in sustainable fuel production from BtX, PtX, or e-/PBtX.

The CES-GIS-SAFAHP methodology uses selected and relevant geospatial data, which is processed to derive criteria-specific datasets, such as spatially resolved energy density maps for biomass-based systems and combined wind and solar energy datasets for hybrid processes. These data are then subjected to a Fuzzy Analytic Hierarchy Process (FAHP), which involves the use of pairwise comparisons and Fuzzy normalization to assign weights to the criteria, ultimately resulting in the generation of weighted overlay maps. The results of both the weighed overlay and a concurrently performed exclusion analysis, delineating areas that fail to meet key conditions or constraints, are combined to produce a final suitability map enabling the identification of optimal plant locations based on their overall suitability index. The proposed approach offers a robust, quantitative framework for spatial optimization in the siting of sustainable fuel production facilities with significant applications for policy-makers, industry, and researchers involved in BtX, PtX, and e-/PBtX scale-up.

The methodology encompasses a comprehensive suitability analysis, …

• Providing a recommended list of suitability and exclusion criteria, categorized into ``requisite,'' ``infrastructure,'' and ``environmental'' criteria, tailored for sustainable fuel production site selection.
• Offering a structured workflow for deriving suitability maps through a combination of GIS-based FAHP with exclusion analysis.
• Providing a practical, replicable algorithm that can guide users through the process, making it easier to apply in various geographic and project contexts.

## Graphical abstract

**Figure:**
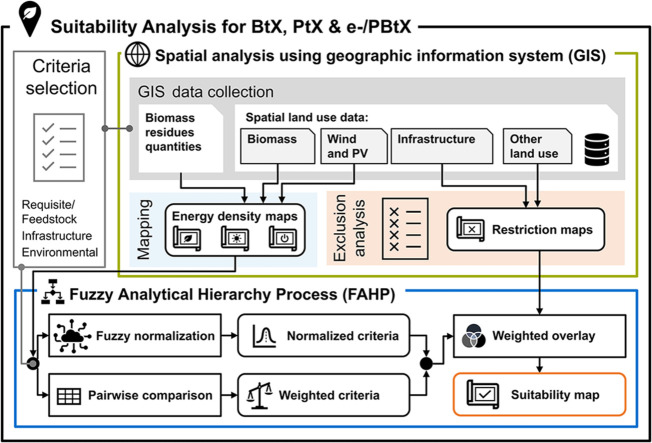

Specifications table

**Table:** 

Subject area:	Energy
More specific subject area:	Suitability analysis, mapping, and site selection to produce sustainable fuels and chemicals from biomass and electricity
Name of your method:	TUM Chair of Energy Systems GIS-based Suitability Analysis using the Fuzzy Analytic Hierarchy Process for optimal BtX, PtX, PBtX, and eBtX plant siting (CES-GIS-SAFAHP)
Name and reference of original method:	Suitability Analysis, Multi-Criteria Decision Analysis, Fuzzy Analytic Hierarchy Process Saaty 1987: https://doi.org/10.1016/0270-0255(87)90,473-8 Jayarathna et al. 2022: https://doi.org/10.1016/j.landusepol.2022.105986 Pfenning et al. 2023: https://doi.org/10.1016/j.apenergy.2023.121289
Resource availability:	N.A

## Background

To reduce energy consumption and mitigate climate change, the transition from fossil fuels to renewable alternatives is crucial. Among these alternatives, biomass residues stand out as key feedstocks for producing high-density fuels, materials, and chemicals. Processes like Biomass-to-X (BtX) and CO_2_ hydrogenation in Power-to-X (PtX) offer sustainable pathways to help defossilizing industries like chemicals, heavy transportation, maritime shipping, and air travel. One novel approach is the electrification of the BtX process, which can double product yields by utilizing biomass residues and renewable electricity [1]. As all these resources have highly uneven spatial distributions, selecting the optimal location for such production facilities becomes critical.

Identifying suitable locations for sustainable fuel production is a complex decision-making process that requires the integration of many factors. Suitability analysis is a common method used in geographic information systems (GIS) to evaluate the appropriateness of a given location or area for a specific purpose. The underlying premise of GIS suitability analysis is that every landscape attribute has inherent characteristics that are either suitable or unsuitable for the planned activities [2]. To consider various factors or criteria that influence the suitability of the location are accessed through the so-called MCDA [3,4]. The resulting suitability map combines these criteria and indicates the degree of suitability of different areas for the intended purpose [5].

Suitability analysis helps decision-makers identify suitable locations for activities such as land development, conservation, agriculture, and infrastructure development. While it is commonly used in urban planning, environmental management, natural resource assessment, and site selection for infrastructure projects [4], there is limited research specifically focusing on the application of MCDA in conjunction with GIS for determining actual plant locations for BtX, PtX or hybrid facilities. While there are studies that use MCDA and GIS for site selection in various contexts, such as renewable energy development [[6], [7], [8], [9], [10], [11], [12]] or environmental management [[13], [14], [15], [16]], the specific application to BtX, PtX or hybrid plant locations is missing.

This study addresses this research gab by proposing a suitability analysis methodology tailored to identify optimal sites for sustainable fuel production. Process-specific suitability and exclusion criteria are proposed, including their recommended application using pairwise comparison and specified fuzzy functions. Using MCDA methods such as FAHP allows the criteria to be normalized and weighted according to their importance using pairwise comparison and Fuzzy membership functions. The combination of Fuzzy normalization of selected criteria with pairwise comparison allows the quantification of normalized and weighted criteria and ensures that decisions are based on a rigorous analysis of the criteria and their relative importance, leading to informed decisions.

Furthermore, an energy density mapping is recommended for biomass-based processes, while separate datasets are proposed for wind and photovoltaic due to spatial resource differences. A combined system is considered as well using the most cost-effective combination. A weighted overlay is proposed to combine the selected criteria. The so-derived suitability map can then be used to identify and prioritize potential plant locations based on their overall suitability scores ranging from 0 to 9 for BtX, PtX, or e-/PBtX processes. Using the proposed suitability analysis, the most suitable location can be identified making it a valuable tool for spatial decision-making, providing a systematic and quantitative approach to evaluating the suitability of locations for sustainable fuel production.

## Method details

The methodology presented in this study serves as a structured approach to systematically identify and assess suitable locations for sustainable fuel production, focusing on Biomass-to-X (BtX), Power-to-X (PtX), and their combinations known as Power-Biomass-to-X (PBtX) and directly electrified BtX (eBtX) [1].

**Fig. 1:**
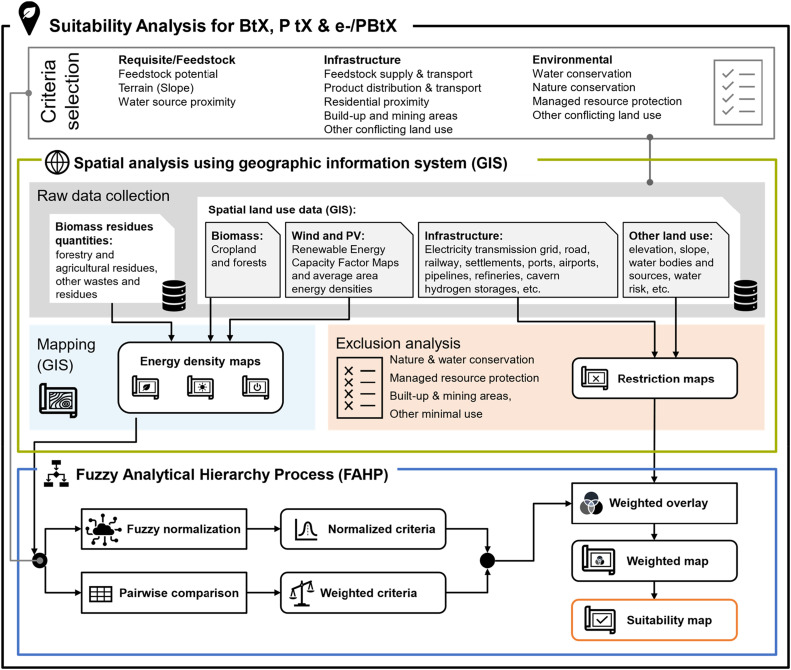
Comprehensive overview of the step-by-step suitability analysis framework using multi-criteria decision analysis (MCDA) to create Biomass-to-X (BtX), Power-to-X (PtX), or hybrid (PBtX, eBtX) suitability maps based on the Fuzzy analytic hierarchy process (FAHP) and geographic information systems (GIS).

As depicted in Fig. 1, the methodology allows for a potential facility site to be evaluated relative to other sites. The methodology begins with the selection of suitability and exclusion criteria, which are tailored to the specific needs of BtX, PtX, and hybrid facilities. Suitability criteria represent factors that positively influence the feasibility of a site for sustainable fuel production, such as feedstock availability or energy resources. A The Multi-Criteria Analysis or Decision Analysis (MCA/MCDA) is used to determine suitable sites and their potential production capacities for sustainable fuel production. The process implements the Fuzzy Analytic Hierarchy Process (FAHP) for data standardization and pairwise comparison. This allows for a structured, expert-driven process to weight these criteria, accounting for uncertainty and producing a weighted suitability map that highlights the most favorable locations. Concurrently, an exclusion analysis eliminates locations that fail to meet essential thresholds (e.g., environmental restrictions or infrastructure availability).

The process of suitability analysis typically involves the following steps [17]:

1. **Objectives definition:** Clearly defining the goals and objectives of the analysis, including the specific criteria that will be used to evaluate suitability.
2. **Criteria selection:** Identifying and selecting criteria that are relevant to the objectives of the analysis. These criteria can include physical, environmental, socio-political, and techno-economic factors and include exclusion as well as inclusion criteria.
3. **Data collection:** Gathering and organize spatial data that represent the selected criteria. This may include data on land use, land cover, topography, soil types, and other relevant factors.
4. **Data standardization:** Standardizing the data to ensure that different criteria can be compared and combined involving normalizing the data to a common scale or unit of measurement.
5. **Criteria weighting:** Assigning weights to the criteria based on their relative importance to enable decision-makers to prioritize certain criteria over others in the analysis.
6. **Overlay analysis:** Combining the criteria layers using GIS overlay techniques to generate a suitability map showing the suitability of different locations based on the weighted combination of criteria.
7. **Map interpretation:** Interpreting the suitability map to identify areas that are most suitable or unsuitable for the intended purpose. This information can be used to make informed decisions about site selection or land use planning.

After objective definition, suitable decision criteria are selected to describe plant location requirements, including feedstock requirements, infrastructure criteria and environmental factors, as well as exclusion criteria that are applied to identify areas unsuitable for plant location due to conflicting land use (see Section 1). Thirdly, spatial analysis using GIS methods is conducted (see Section 2). This involves comprehensive feedstock data collection and spatial organization. Data processing is employed to allow process-specific spatial analysis of biomass residues and renewable power. Selected criteria layers are normalized to a common scale, typically ranging from 0 to 1 (see Fuzzy Normalization), to facilitate comparison (see Pairwise Comparison) and aggregation of criteria (see Weighted Overlay and Suitability Mapping), producing a suitability index for each site. This proposed methodology shown in Fig. 1 follows these steps for the BtX, PtX, e-/PBtX specific suitability analysis as summarized in the attached Electronic Supplementary Information (ESI) Section S1 in the form of simplified workflow algorithms for BtX and PtX.

## Criteria selection

In suitability analysis, criteria are used to structure the classic multiple-criteria evaluation problem, prioritize alternatives, and ultimately make an informed decision based on a comprehensive assessment of these criteria. Decision criteria are specific attributes, characteristics, or requirements used to evaluate and compare alternatives in a decision-making process. In GIS-based spatial analysis, such criteria are often represented as spatial data layers with values that can be quantified, such as continuous raster layers or discrete thematic layers. Furthermore, exclusion criteria are criteria that are used to identify and exclude unsuitable locations that do not meet certain minimum requirements or that pose significant risks or challenges (see Recommended Exclusion Criteria). By using both inclusion and exclusion criteria, decision-makers can ensure that only suitable locations are considered for sustainable fuel production plants, while avoiding sites that may be problematic or undesirable. The present study aims at providing a comprehensive selection process, where the chosen criteria aim to ensure a robust framework for evaluating potential locations, balancing the technical, economic, and environmental aspects essential for sustainable fuel production.

### Process-specific consideration

To identify suitable locations for fuel production sites from BtX, PtX, and their combinations (PBtX, eBtX), criteria that are critical to the success and sustainability of a future plant must be selected for the decision-making processes (see Fig. 1). The selection of these criteria is grounded in a systematic approach, which incorporates both empirical data and theoretical frameworks relevant to biomass conversion technologies. Criteria discussed in this study are refined through discussions with academic and industry experts, ensuring that they are not only theoretically sound but also practically applicable in real-world scenarios. This collaborative process allows the identification of attributes that accurately reflect the operational requirements and sustainability considerations essential for effective fuel production site evaluation.

**Fig. 2:**
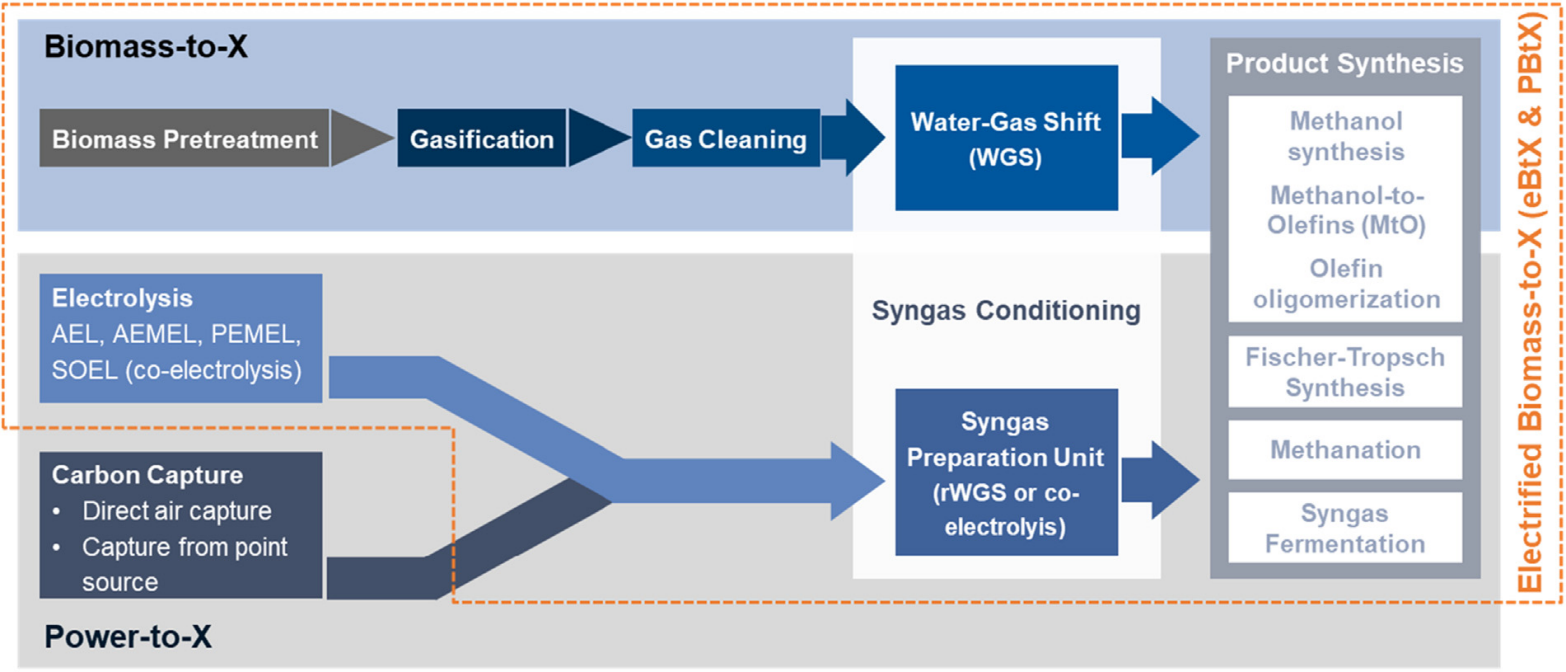
Simplified block diagram of BtX, PtX and e-/PBtX processes based on [20].

To evaluate the quality of potential plant locations, the general process options must be understood. This methodology paper focuses on processes converting solid lignocellulosic biomass and/or renewable electricity (RE) to 2nd generation fuels or chemicals as shown in Fig. 2. The process includes biomass pretreatment, gasification, syngas conditioning, and fuel synthesis [18]. Final products are primarily in liquid form, such as Fischer–Tropsch (FT) products, methanol (MeOH), and other alcohols. Synthetic natural gas (SNG) is a possible gaseous product of the process [1].

Fig. 2 also shows the PtX pathway to produce the same products. Here, typically water electrolysis and a sustainable CO_2_ source are combined to produce syngas which can be used in synthesis [19]. Electrification of BtX processes is defined as the use of additional RE to enhance fuels and chemical production. The term Power-and-Biomass-to-X (PBtX) is typically used for indirect electrification (mostly H_2_ addition), and directly electrified BtX (eBtX) for directly electrified processes [1]. The criteria discussed below are chosen based on previous studies [6,7] as well as through discussions with academic and industry experts.

Based on this process framework, several criteria can be prioritized to enhance decision-making and the viability BtX, PtX, and e-/PBtX. The success of any fuel producing process fundamentally relies on the consistent and adequate availability of suitable feedstocks, which includes both sustainable biomass and renewable energy sources. The availability of these feedstocks also determines the scale of the process, influencing the quantitative requirements for additional feedstocks, utilities, and labor. Similarly, access to abundant, clean freshwater sources that do not compete with human or animal needs is essential, making this a requisite criterion.

Infrastructure and logistics are also critical to the economic viability of fuel production; proximity to feedstock sources and well-developed transportation networks can significantly reduce costs and time. Robust distribution networks for final products facilitate market connectivity, ensuring efficient delivery to consumers. Environmental impact and competing land use are additional crucial considerations for sustainable fuel production, as they help maintain ecological integrity and prevent habitat destruction. A thorough assessment of these impacts ensures that production activities do not compromise food security or essential agricultural practices. Furthermore, addressing land use conflicts is vital for regulatory compliance and public acceptance, as stakeholders increasingly prioritize sustainability.

Feedstock, infrastructure, and environmental criteria are interlinked and play a crucial role in determining the feasibility, efficiency, and sustainability of fuel production initiatives. Quantifying and weighing these criteria against one another is essential for informed decision-making and improving the likelihood of project success. Therefore, these criteria are systematically analyzed and discussed in the following sections.

### Requisite/feedstock criteria

For future (P-/e)BtX plants, a reliable supply of sustainable feedstock is crucial. Biomass, RE, and water are therefore the most important resources that must be available in sufficient quantities on site. Furthermore, feedstock availability is an important economic criterion to consider with biomass feedstock costs accounting for about 40 % of the BtX levelized cost of production. For PtX processes electricity costs also make up around 70 % of the total expenses, while for PBtX processes, electricity costs account for about 30–40 %, and biomass feedstock costs for about 25 % of total cost [21].

BtX processes use non-edible, lignocellulosic biomass, and its residues, such as crop residues, grasses, sawdust, wood chips, or sewage sludge, to be converted into syngas via gasification as shown in Fig. 2. Such Biomass must be sustainably sourced to achieve the goal of low environmental impact products. Biomass availability strongly depends on the geographic location and can either be sourced locally, regionally or transported to the plant from a greater distance [20].

For PtX and P-/eBtX processes, the availability of RE is comparable to that of biomass for BtX. While technically, for PtX and PBtX plants, the required green hydrogen could be produced off-site and transported to the plant's location [1], in this methodology we focus on plants, requiring sufficient RE supply on-site at any given time. RE must thus be available nearby or delivered via the high-voltage transmission grid, potentially with sufficient electricity storage capacity to compensate for volatile wind and photovoltaic (PV) power.

The suitability of (P-/e)BtX plants depends on their geographic location in relation to feedstock locations and quantities. While transporting issues are covered by Infrastructure Criteria, the biomass and electricity availability is chosen as requisite criterium. Spatial energy density is selected as a criterion for biomass and RE availability as it allows for a comprehensive evaluation by combining both the quantity and spatial distribution of biomass and RE sources. For biomass, energy density is particularly useful because the quantity of biomass in terms of mass may not directly correlate with the scale of a BtX plant. Energy density provides a more accurate measure for comparing different locations based on their biomass potential. Similarly, for RE sources like wind and PV, energy density is essential for comparing locations beyond just their capacity factor. However, it's important to note the challenges posed by conflicting use of wind and PV, which can complicate the assessment of energy density.

The terrain slope is also included as a requisite criterion, as too steep areas can pose challenges for various aspects of P-/eBtX plant operations. Steep terrain can make the construction and maintenance of wind and PV installations more difficult and costly. It can also impact the harvesting and collection of biomass residues, as well as the construction of (P-/e)BtX plants, potentially leading to inefficiencies or safety issues.

A reliable supply of water is also essential for the practical operation of (P-/e)BtX plants, as it water plays a crucial role as a process utility as well as in hydrogen production through water electrolysis. The electrolysis process splits water molecules into hydrogen and oxygen using electricity, with the hydrogen being used as a primary feedstock for the synthetic fuel production. Potential water sources can vary depending on the plant's location and can include sources such as freshwater, desalinated seawater, or recycled wastewater [7]. To ensures sufficient water supply, water source proximity is selected as a requisite criterium. While water quality is subjected to the quality of the GIS data source, the environmental compatibility is covered by an environmental exclusion criterium below.

### Infrastructure criteria

The availability of feedstock and the terrain characteristics are interdependent and can sometimes conflict with each other. Considering additional logistical aspects is crucial for ensuring the efficient operation of the plant. Transportation infrastructure plays a critical role in the overall supply chain as it must be able to support the transportation of raw materials to the plant and the distribution of products to the market.

Transportation cost can significantly impact the overall cost structure of (P-/e)BtX plants. For BtX and electrified BtX, biomass can be sourced locally, regionally, or transported from a distance, using either decentralized or centralized pretreatment. Typically transport costs are a main contributor to BtX levelized cost of production [22] making locally-sourced biomass more eligible in terms of transport economics as well as sustainability. Road, rail, or ship transportation are necessary for biomass, while transmission lines are essential for electricity supply. Availability of product distribution networks play an important role on the demand side. For products such as fuels and chemicals, that typically includes oil and gas infrastructure such as pipelines, refineries, but also ports and potentially airports. Thus, to quantify infrastructure as a GIS criterion, the distance to transportation networks is used. Here, the proximity of each potential plant site to the nearest road, rail, and waterway is selected as preference criterium in accordance with Jayarathna et al. [6] and Pfennig et al. [7]. Large-capacity hydrogen storage sites like suitable salt caverns might also play a special role in boosting PtX and PBtX projects in the future.

Furthermore, including the availability of skilled labor and residential proximity as criteria for large-scale (P-/e)BtX projects is crucial [7]. By focusing on larger cities, where skilled labor is more readily available, the feasibility of such projects can also be improved. Additionally, proximity to residential areas can facilitate the transportation and distribution of products. This approach not only considers the technical aspects of the projects but also the social and economic factors that contribute to their success.

By excluding specific areas with existing infrastructure, it can be ensured that the selected sites are free from potential conflicts and can support the development of P-/eBtX plants without interference from other activities. Including criteria related to existing infrastructure such as built-up areas, mining sites, defence land, and stock routes is essential for identifying unsuitable plant sites. Using proximity criteria for these factors allows for a quantitative assessment using GIS, making it easier to identify suitable sites based on these criteria (see Recommended Exclusion Criteria).

### Environmental criteria

In suitability analysis, areas with a particularly high potential for sustainable fuel production are to be identified where the use of the specific renewable technology is not expected to have a significant impact on the environment. Thus, including environmental criteria in the assessment of potential plant sites is crucial for ensuring sustainable and ecological operations. In general, in the construction stage of fuel producing facilities, grid connection infrastructure and access roads, land clearance is required, which may lead to the deterioration or loss of habitats. The operation of such plants may result in further negative environmental impact which needs to be mitigated. The impacts vary depending on the previous use of the area and technology employed. Spatial accumulation of renewable energy projects, for example, can lead to such structural impacts as habitat fragmentation, degradation or loss, barrier effects and population shifts, as well as pollution generated by construction and maintenance operations, and an increased risk of wildlife injury and mortality [23].

By considering the proximity to lakes, rivers, wetlands, and other protected areas, the potential impact of any (e-/P)BtX plant on these sensitive ecosystems can be minimized. Managed resource protection criteria further contribute to the conservation of biodiversity and natural resources. Incorporating these ecological criteria alongside technological considerations helps ensure that the selected plant sites are not only feasible from a technical standpoint but also environmentally responsible [7].

The environmental criteria are mainly used to exclude unsuitable locations that pose significant risks to the environment (see Recommended Exclusion Criteria).Thus, the distance to water and nature conservation including lake, river, marsh/wetland, estuary/coastal waters conservation proximity as well as strict nature reserve, wilderness area, nature park, natural feature protection, habitat/species management area, protected landscape, other conserved area proximity are included. Also managed resource protection can be considered in the form Biodiversity, surface water supply, groundwater, landscape, traditional indigenous uses. Other minimal use criteria include natural areas, residual native cover, rehabilitation.

### Recommended suitability criteria

The challenge in the final criteria selection for suitability analysis is selecting the most important decision criteria that must be available and quantifiable to allow the application of mathematical methods, such as weighting and Fuzzy normalization, to combine the criteria and generate a suitability map for each alternative. While the criteria availability depends on the GIS data availability, selecting the key criteria from the vast number of factors can be challenging. Based on literature review, expert consultation, stakeholder engagement, and the discussions above, a preliminary list of criteria is refined by eliminating criteria that are redundant or less relevant and prioritizing criteria that are critical for the site selection process. Where possible, the selected criteria are quantified to make them measurable and comparable. The final criteria are considered comprehensive, relevant, GIS-compatible, and measurable. The resulting short list of criteria being relevant to the (e-/P)BtX plant site selection process is provided in Table 1.

**Table 1:** Short-list of recommended suitability criteria to enable decision-making in suitability mapping of BtX PtX and e-/PBtX plants. (specific for a—BtX processes; b—PtX processes; c—e-/PBtX processes).

Requisite (Feedstock criteria)	Infrastructure	Environmental
*Feedstock potential* (Biomass^a,c^, Wind and PV^b,c^ spatial energy density) *Terrain* (Slope) *Water source proximity*	*Feedstock supply & transport* (road & railway proximity, electricity transmission line proximity^b,c^) *Product distribution & transport* (Oil pipeline proximity, refinery proximity, Port & Airport proximity) *Hydrogen storage proximity*^b,c^ *Residential proximity* *Build-up areas* (Services building, manufacturing, and industrial building) *Mining areas* (Mines, quarries, tailings, extractive industry not in use) *Other conflicting land use* (Defence land, stock route)	*Water conservation* (Lake, river, marsh/wetland, estuary/coastal waters conservation proximity)
		*Nature conservation* (Strict nature reserve & park, wilderness area, natural feature protection & protected landscape, habitat/species management & other conserved area) *Managed resource protection* (Biodiversity, surface water supply, groundwater, landscape, traditional indigenous uses) *Other conflicting land use* (natural areas, residual native cover, rehabilitation)

### Recommended exclusion criteria

Additional site selection-related challenges other than environmental considerations, such as conflicts with other existing land uses, or legal minimum distance requirements to housing, which in some cases can make most of the territory of a country inaccessible for installing renewable energy plants. Conflicting land use that occurs when a piece of land can be used only for one use or the other. Therefore, finding out if any obstacles or restrictions exist for constructing the plant, collecting biomass residues, and/or installing the planned renewable energy technology is a key step in the broader area selection process [23]. Carrying out this exclusion analysis at an early stage allows time for any potential amendments to laws or regulations, where these may be necessary. Thus, it is crucial to take into account the regulations pertaining to environmentally, geographically, and culturally significant protected natural and man-made areas. Based on the exclusion analysis by Jayarathna et al. [6], exclusion criteria are defined as part of a criteria catalog above and summarized in Table 2 as a list of types of land use area that are to be excluded from this analysis.

**Table 2:** List of recommended exclusion criteria in suitability mapping of BtX PtX and e-/PBtX plants.

Criteria type	Restrictions	Specification
Environmental	Nature conservation	Strict nature reserve, wilderness area, nature park, natural feature protection, habitat/species management area, protected landscape, other conserved area
	Managed resource protection	Biodiversity, surface water supply, groundwater, landscape, traditional indigenous uses
	Water conservation	Lake, river, marsh/wetland, estuary/coastal waters conservation
Infrastructure	Build-up areas	Services building, manufacturing and industrial building
	Mining areas	Mines, quarries, tailings, extractive industry not in use
	Other minimal use	Defence land - natural areas, stock route, residual native cover, rehabilitation

Table 2 includes both natural restricted area and intensive human activity area. These restricted areas are deemed as unsuitable in the context of this study and would be excluded from the analysis. In addition, a buffer zone of 1 km for each type of land use area is created to steer clear of restricted area. The exclusion analysis described in Section 6 involves overlaying the exclusion criteria map layers identifying areas that meet the exclusion criteria to mark them as unsuitable for (e-/P)BtX plant sites [6,7,24].

## Feedstock GIS data processing

While GIS information on terrain slope and water sources is generally available for most locations globally, the spatial distribution data on feedstock availably not only depends on the quality of GIS data in different regions and countries, but typically also requires some adjustment to be used in suitability analysis.

### Biomass residues

For the employed biomass-based process routes, biomass residues are the only carbon-neutral feedstock option. When selecting a feedstock for a BtX plant, biomass characteristics and availability is subject to seasonal and geographical variations and multiple residues present themselves as potential sources, including forestry and agricultural residues, as well as other wastes. Potential forestry residues, include primary residues from e.g. logging and other pre-commercial thinning, as well as secondary residues, such as woodchips and pellets, sawdust and black liquor. Primary agricultural residues for BtX applications mainly consists of dry and wet manure, while secondary residues cover e.g. olive pits. Solid agricultural residues including waste from the cutting of permanent crops as well as straw and stubble residues. Other waste biomass sources include residues from landscape care management, roadside greens and abandoned lands, as well as biomass residues from different industries, sewage sludge and municipal solid waste [25].

Accessible data regarding the availability of lignocellulosic biomass residues have different format and display which makes data processing unavoidable. One challenge with biomass spatial GIS data is that it often comes from sources such as national or regional statistics, which may aggregate data to larger geographic units for reporting purposes. Being lumped for regions or areas, results in low spatial resolution meaning that the data does not provide detailed information on the distribution of biomass residues within a given area, making it challenging to accurately assess biomass availability at a finer scale. This lack of detail can be problematic when trying to identify suitable locations for biomass-to-liquid plants, as it may lead to inaccuracies in estimating the quantity and spatial distribution of biomass resources.

**Fig. 3:**
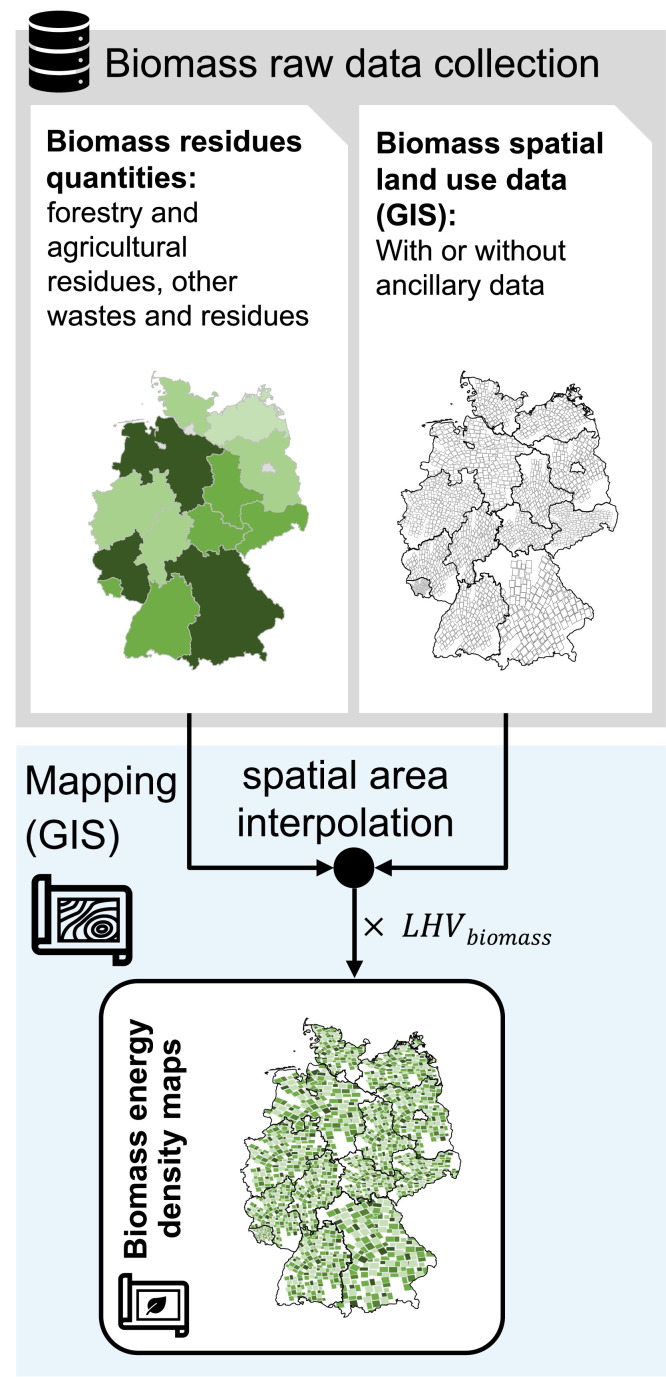
General Methodology for biomass energy density mapping using GIS data, spatial area interpolation and lower heating value of biomass (LHV_biomass_).

There are several methods to redistribute lumped biomass residue data from larger areas, such as municipalities, to higher-resolution areas like farmland. In geographical research, spatial area interpolation is a widely applied method that uses sample values of known geographical area units to estimate values at other unknown area units resulting in values at finer resolutions than the original data [26]. As shown in Fig. 3, this approach helps to create high-resolution biomass maps that reflect the actual distribution of biomass resources on the ground.

Spatial area interpolation methods can be classified according to whether they are based solely on the characteristics of the source and target areas or whether they use additional data to make a more informed interpolation [26]. The choice of approach is often dictated by the availability of relevant datasets. Area-weighted interpolation is the simplest and most effective method allocating biomass quantities from source zones proportionately to target zones based on the area of their intersection. The method is straightforward to implement using polygon overlay operations where area weighting is inherently volume-preserving [26]. However, a significant limitation of this method is its assumption of a spatially homogeneous relationship between the source zone attribute and the target zone areas [27]. Nonetheless, in the absence of ancillary data, area weighting remains a reasonable solution.

In contrast, when ancillary information with a relevant relationship to the source zone variable is available, it can be used to constrain or inform the allocation to target zones. Approaches for areal interpolation of biomass residues utilizing ancillary data include statistical or point-based approaches like kernel density mapping, as well as or dasymetric mapping, with the latter being the most commonly used method []. Here, for example, satellite or aerial imagery can be used to assess vegetation health and cover by measuring the difference between near-infrared reflectance, thus providing valuable information about vegetation density to inform the areal interpolation process. Hence, dasymetric mapping can improve the spatial resolution of biomass resource maps by using land cover data, which provides information on the types and locations of different land cover classes (e.g., forests, croplands, grasslands) [28,29].

Given the diverse distribution of various residue types, combining them into a unified spatial distribution in terms of energy density as shown in Fig. 3 is a recommended approach. For each type of biomass residue, existing GIS data is to be converted into a potential energy spatial distribution in terms of GJ_LHV,biomass_ per area and time using the respective mean calorific values. Subsequently, the computed rasters are merged through addition, resulting in a consolidated map depicting the potential biomass energy density.

### Renewable energy

For electricity-based process routes, electricity availability and spatial distributions is crucial. Since any employed electricity needs to be 100 % renewable, geothermal, hydro, biomass, wind, and PV are the only potential sources. As electricity generation from biomass is inefficient, biomass should be used for BtX processes rather than power generation. With hydro-electricity and geothermal potential being globally limited, PV and wind are the most promising sources for a massive scale-up of RE for PtX and e-/PBtX applications. In accordance with the EU's delegated act on green hydrogen, only electricity that is directly supplied from a wind or PV source to the plant is considered fully renewable (direct sourcing). While it is possible to connect the RE source and the SAF production plant via the grid, this connection must be facilitated through a smart metering system to trace the origin of the electricity consumption. Furthermore, the electricity source must meet the additional condition that it must be specifically constructed for the purpose of operating the PtX or e-/PBtX plant. This is intended to prevent the utilization of pre-existing renewable electricity capacity for hydrogen production, which could indirectly increase the demand on fossil-fueled power plants for electricity supply [30].

**Fig. 4:**
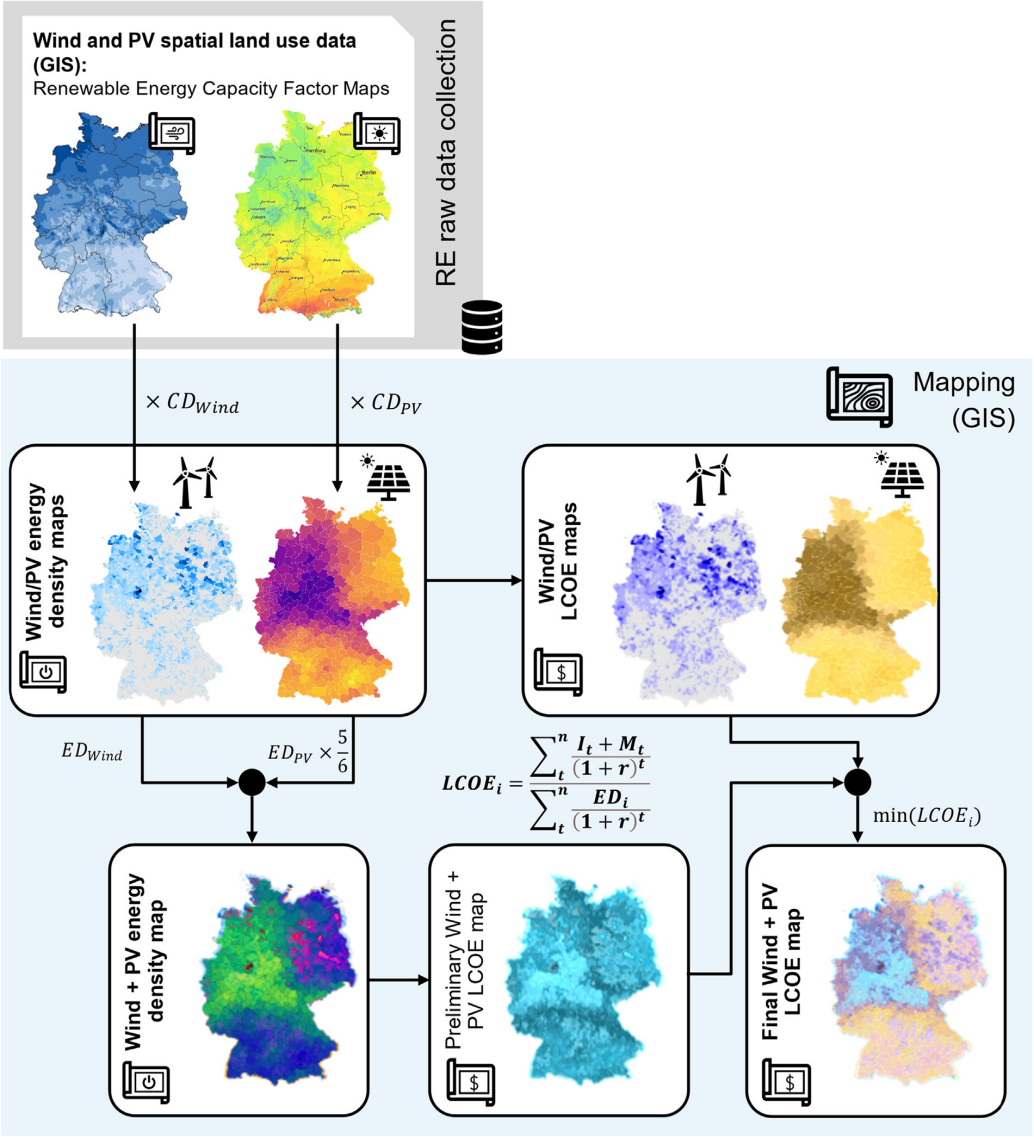
General Methodology for renewable electricity generation (Wind, PV) energy density mapping using GIS capacity factors and Levelized Cost Of Electricity (LCOE) calculations for the combined system.

To evaluate the technical potential of wind and PV power, spatial datasets provide specific location-based capacity factor maps both for wind and PV. These values are typically based on spatial analysis combined with high-resolution mapping of meteorological data, offering an insight into the potential energy production relative to the installed peak capacity. However, similar to biomass, the actual achievable energy density presents a better metric to compare different wind and PV sites. Converting capacity factor maps for wind and PV into potential energy density maps involves multiplying the installed capacity density CD for wind and PV by the capacity factor for each location as shown in Fig. 4. The result is the maximum potential energy density at each location, expressed in GWh_el_/km², according to:

(1) $$ED_{Wind/PV,i}=CF_{Wind/PV,i}\cdot CD_{Wind/PV}\cdot 8760\;h/a$$

where $ED_{Wind/PV,i}$ is the potential wind or PV energy density of the *i*th grid of the map in MWh_el_/km^2^/a; $CF_{Wind/PV,i}$ is the wind or PV capacity factor of the *i*^th^ grid; $CD_{Wind/PV}$ is the installed capacity density for wind or PV in MW_el_/km^2^. However, obtaining the installed capacity density for wind and PV involves several considerations and challenges, as the installed capacity density varies based on the location's suitability for wind or solar energy generation. Factors such as wind speeds, solar irradiance, and available land area influence the optimal density. Also, different wind turbine models and solar panel technologies have varying efficiency and capacity factors. The choice of technology affects the installed capacity density.

Estimating the installed capacity density requires assumptions about the size and spacing of wind turbines or solar panels. These assumptions can vary based on the specific technology and site characteristics. While detailed methods for the selection of the most efficient and cost-effective energy solutions based on the available wind/solar irradiation resources and site characteristics exist [7,25], in this method we propose the use of average installed capacity densities as a practical approach for broader-scale assessments. The suggested average installed capacity density for PV is 30 MW_el_/km^2^, while for wind the installed capacity density is estimated to be 2.8 MW_el_/km^2^ [31].

This study also considers the possibility of a combination of PV and wind systems at the same location. A good general estimate for the installed capacity density of PV in a combined PV and wind system, when avoiding detailed analysis, should consider the need to balance the available area for PV installation with the presence of wind turbines. As wind turbines typically require larger spacing between them to avoid wake effects, it is assumed that PV would cover the space in between. Due to the reduced available area for PV installation, the installed capacity density for PV in the combined system is assumed to be reduced by one sixth. The energy density map for combined PV and Wind power generation is calculated as follows:

(2) $$ED_{Wind+PV,i}=\left(CF_{Wind,i}\cdot CD_{Wind}+CF_{PV,i}\cdot CD_{PV^{*}}\right)\cdot 8760\;h/a$$

where $ED_{Wind+PV,i}$ is the potential energy density from the hybrid system of the *i*^th^ grid of the map with the unit of MWh_el_/km^2^/a; and $CD_{PV^{*}}$ is the potential PV capacity density of the *i*^th^ grid being reduced to a value of 25 MW_el_/km^2^. While the combined energy density mapping for a PV and wind system can provide insights into maximizing energy potential at a potential site, it does not directly account for the costs of electricity generation.

The cost-effectiveness of each technology is a crucial factor to consider in addition to the energy output. The investment cost $I_{t}$ and operation and maintenance cost $M_{t}$ of wind and PV systems differ, which can significantly impact the overall cost of the electricity produced. The Levelized Cost Of Electricity (LCOE) is a suitable standard metric that represents the per-unit cost of generating electricity over the lifetime of a technology, taking into account all costs and energy production, as defined in Eq. (3) [32].

(3) $$LCOE_{i}=\frac{\sum_{t}^{n}\frac{I_{t}+M_{t}}{{\left(1+r\right)}^{t}\;}}{\sum_{t}^{n}\frac{ED_{i}}{{\left(1+r\right)}^{t}\;}}$$

where $LCOE_{i}$ is the LCOE of the *i*th grid of the map, t the year of operation, n the lifetime of the technology, r the interest rate, and $ED_{i}$ is the potential energy output of the *i*^th^ grid from the map. A 7 % interest rate and a uniform lifespan of 20 years for all technologies is assumed for LCOE calculations.

The cost data used for wind and PV installation and operation is summarized Table 3. Thus, as shown in Fig. 4, LCOE maps for PV, wind, and the combined systems can be generated. A comprehensive potential renewable energy LCOE map for renewable energy is created by selecting the minimum LCOE for each cell. This derived map serves as decision criterion for the suitability analysis of the e-/PBtX process based on the optimal combined availability and cost of renewable electricity from PV, wind, or both.

**Table 3:** Assumptions for renewable energy mapping: capacity density, investment and operation & maintenance (O&M) costs for different renewable energy technology from [33].

		Investment cost $I_{t=1}$	O&M cost $M_{t}$	Capacity Density CD
		$_2022_/kW_p_	$_2022_/kW/a	MW/km^2^
PV	Stand-alone	923	7.4	30
	combined	923	7.4	25
Wind	onshore	1361	30	2.8
	offshore	3461	92 [34]	2.8

## MCDA methodology and tools

The presented suitability analysis aims to establish criteria-based mapping integrating spatial data and analysis techniques. To meet the needs of many stakeholders, systematic decision-making frameworks such as MCDA to support more informed and spatially explicit decision-making processes are a good approach. The origins of Multi-Criteria Analysis (MCA) and Multi-Criteria Decision Analysis (MCDA) go back to the concepts of decision calculus, developed in the 1950s [[35], [36], [37]]. In the 1970s and 1980s, the field was expanded to decision analysis and multi-attribute utility theory [[38], [39], [40], [41], [42]]. These methodologies provide frameworks for structuring decision problems involving multiple criteria and uncertainties. The formalization and widespread application of MCA and MCDA methodologies in various fields, including environmental management, engineering, and public policy, have been the result of contributions from a large number of researchers and practitioners over the years [[43], [44], [45], [46], [47], [48], [49], [50]]. While MCDA is generally used to evaluate and compare alternatives based on multiple criteria or objectives, considering qualitative and quantitative factors, GIS-based methods, involve the use of spatial data and analysis techniques to analyze geographic information. The use of GIS in combination with decision analysis methods, including MCDA, has become increasingly common in environmental and resource management studies [[51], [52], [53], [54]] over the past few decades.

The methodology presented in this paper uses MCDA methodology alongside GIS-based methods to assess suitable locations for sustainable fuel production. Since this plant siting is a classic multi-criteria evaluation problem, which involves evaluating a set of finite alternatives based on multiple criteria to determine the most suitable option, a variety of MCDA methods are available. The Analytic Hierarchy Process (AHP), Fuzzy AHP (FAHP), Technique for Order of Preference by Similarity to Ideal Solution (TOPSIS) [50], or even more advanced methods like Elimination and Choice Expressing Reality (ELECTRE) [45] or Preference Ranking Organization METHod for Enrichment Evaluations (PROMETHEE) [46] allow to compare alternatives based on their performance across multiple criteria and derive a ranking or classification.

Here, the FAHP is selected as MCDA method because it is a particularly well-suited structured approach to compare and prioritize selected criteria based on their relative importance. The AHP was first developed by Thomas L. Saaty [43]. Developed as a decision-making framework to deal with complex problems involving multiple criteria and alternatives, the method helps to structure the problem into a hierarchy, comparing the importance of criteria and alternatives. AHP has been widely used in various fields, including business, engineering, environmental management, and healthcare, among others. It has also found extensive application as a decision-making tool in the assessment, choice, and strategic development of energy systems that encompass both renewable and non-renewable energy sources [55].

In FAHP, pairwise comparison is employed to help determine the relative importance of criteria, while Fuzzy normalization is used to handle the imprecision and uncertainty inherent in decision-making processes. A consistency check is performed to ensure that the pairwise comparisons made by decision-makers are logical and consistent. Normalized Fuzzy comparison matrices are aggregated to obtain the overall priority or weight of each criterion and alternative. The combination of both methods a systematical comparison of all selected criteria by establishing their weights, which can then be used to evaluate and rank potential plant locations. This ensures that the final decision is well-balanced and considers all relevant factors. Moreover, FAHP can facilitate stakeholder engagement by providing a transparent and structured approach to decision-making. Stakeholders can participate in the FAHP process by providing their input on the relative importance of criteria, which can help build consensus and ensure that the final decision is acceptable to all parties involved.

### Pairwise comparison

**Fig. 5:**
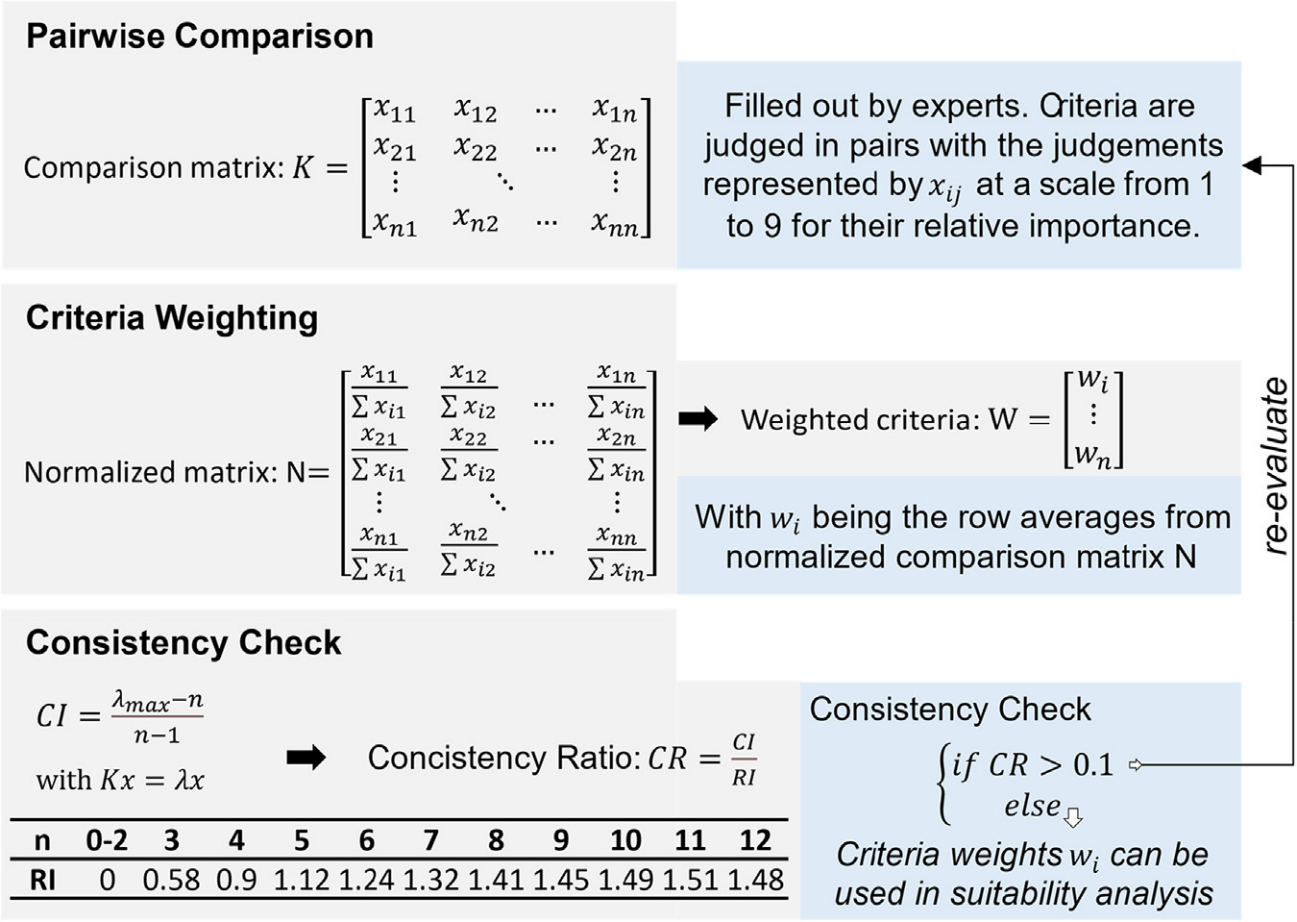
Pairwise comparison methodology and calculations applied in the analytic hierarchy process for suitability analysis.

To generate a unified suitability map, all criteria must be combined through a weighted overlay. Comparing the relative importance between different criteria is used to assign a weight to each criterion. Introduced by Saaty, the concept of pairwise comparison to systematically compare and prioritize criteria and alternatives in decision-making processes [43], it is now frequently employed as the primary method for determining criteria weights in GIS-MCDA applications [51,56]. Here, the criteria are judged in pairs with the judgements represented by a scale with values from 1 to 9 for their relative importance [43]. Thus, a pairwise comparison matrix can be generated as shown in Fig. 5 allowing the criteria to be weighted against each other [6]. Given a comparison matrix K of a size of n×n, with n being the number of compare criteria, its element $x_{\mathrm{ij}}$ denotes the relative importance with a value ranging from 1 to 9 [43]. The criteria weights are obtained from the average of each row of the normalized matrix.

In the FAHP pairwise comparison, there is no strict limit to the number of criteria. However, as the number of criteria increases, the complexity of the analysis also increases, making it more challenging to ensure consistency and meaningful comparisons [57]. It is generally recommended to keep the number of criteria manageable and relevant to the decision-making process. Too many criteria can lead to decision-making difficulties and may reduce the reliability of the results.

To measure the degree of uncertainty of subjective judgement, a Consistency Ratio (CR) is defined to check the matrices for inconsistency. The purpose of the CR is to ensure that the judgements made in the pairwise comparisons do not contradict each other. CR is calculated based on the Eigenmatrix of the pairwise comparison matrix K yielding the Consistency Index (CI), as well as a Random Index (RI). If the CR value is smaller than 0.1, the weights assigned to the criteria are satisfactory. Otherwise, the subjective judgements should be revised [43].

CI is calculated as the difference between the maximum eigenvalue $\lambda_{\text{max}}$ of K and its order (n), divided by (n−1) (see Fig. 5). CI provides a measure of how much the pairwise comparisons deviate from perfect consistency. However, since the absolute value of CI depends on the matrix size, it needs to be normalized by RI, which is derived from empirical studies and is used as a reference value to assess the consistency of pairwise comparison matrices. It represents the average consistency index of a randomly generated pairwise comparison matrix. The RI values are pre-defined based on the size of the matrix (the number of criteria or alternatives being compared). For n×n matrices with n<3, RI is 0. For more criteria compared, Fig. 5 provides the respective RI up to a maximum recommended number of 12 criteria. CR can then be calculated as the ratio of CI and RI as defined in Fig. 5. [43]

To illustrate the general methodology, an exemplary pairwise comparison was conducted. Based on reported expert opinions for SAF producing BtX, PtX, as well as hybrid e-/PBtX processes (see Fig. 2) pairwise comparison matrices are derived, and criteria weights are calculated. Pairwise comparison can be a useful tool for selecting the most important criteria from a long list of possible suitability or decision criteria by starting with a larger number of criteria then actually recommended and then eliminating the less important criteria based on the resulting weighting. This can be particularly helpful when there are many criteria to consider, and it is necessary to focus on those that have the greatest impact on the suitability analysis. However, since too many criteria can lead to inconsistencies and difficulties in deriving meaningful weights, it is generally recommended to start with a manageable number of criteria that cover the most important suitability aspects. As shown in Fig. 5, such pairwise comparison is often an iterative process, where criteria weights and data layers may be adjusted based on feedback and further analysis. The resulting list of criteria include the 9–10 most important suitability criteria when evaluating the siting of BtX, PtX, and e-/PBtX plants. CR are used to ensure consistency showcasing the legitimacy of the resulting weighting.

### Fuzzy normalization

Although AHP is widely used, it faces a challenge in its inherent incapacity to handle the imprecision and subjectivity associated with human judgment and the uncertainties inherent in decision criteria. While in simple pairwise comparison each GIS data set in each cell is considered either suitable or not, Fuzzy logic is specially designed for situations in which the boundaries between classes are not clear. However, it is possible to resolve the interpretation of linguistic terms or uncertainty measures of the AHP through Fuzzy logic [58]. Fuzzy set theory, which forms the basis of FAHP and Fuzzy normalization, was developed by Lotfi A. Zadeh in the 1960s. Zadeh, a mathematician and computer scientist, introduced the concept of Fuzzy sets to represent and work with uncertainty and vagueness in decision-making and artificial intelligence [59]. The application of Fuzzy normalization specifically in the context of FAHP and decision-making processes has been further developed by researchers in the field of decision science and Fuzzy logic. The use of Fuzzy normalization in FAHP has become a valuable technique for handling uncertainty and imprecision in decision-making.

To use FAHP in suitability analysis, the Fuzzy membership or transformation function u(x) is employed converting the GIS data into a scale ranging from 0 to 1, determined by the suitability. A value of 0 is assigned to locations that are unquestionably not suitable, while a value of 1 is assigned to those that are very suitable, typically the maximum values in a GIS data set. All values between 0 and 1 are allocated to various degrees of potential membership [60].

Examples of Fuzzy transformation functions include triangular, trapezoidal, and Gaussian membership functions. The Fuzzy transformation functions used in this paper, are either of the “linear” type, or of the type “small”, “large”, or “large and small”. The Fuzzy linear transformation function is applied with the given minimum and maximum values. As shown in Eq. (4), anything below the minimum will be assigned a 0 (definitely not a member) and anything above the maximum a 1 (definitely a member). Anything in between is assigned to a value according to a linear function with a constant slope [60].

(4) $$\begin{array}{c} u{\left(x\right)}_{\text{linear}}=\left\{ \begin{array}{cc} 0 & \text{if}\;x\leq \text{min}\\ \frac{x-\text{min}}{\text{max}-\text{min}} & \text{if}\;\text{min}<x<\text{max}\\ 1 & \text{if}\;x\geq \text{max} \end{array}\right. \end{array}$$

The Fuzzy “large” transformation function $\mu {\left(x\right)}_{\text{large}}$ is applied when larger input values are deemed to have a higher likelihood of being part of the set. A specified midpoint serves as a reference point, receiving a membership value of 0.5. Values exceeding this midpoint are more inclined to belong to the set, while those falling below it exhibit progressively diminishing membership. Vice versa, the Fuzzy small transformation function $\mu {\left(x\right)}_{\text{small}}$ is used when smaller input values are more possible to be part of the set. The spread parameter determines the characteristics and shape of the transition zone. Larger spread means a steeper transition zone. Eq. (5) describes the Fuzzy large function and Eq. (6) the Fuzzy small function. The Fuzzy large and small function is the product of both Fuzzy large and small function [60].

(5) $$\begin{array}{c} u{\left(x\right)}_{\text{large}}={\left[1+{\left(\frac{x}{f_{2}}\right)}^{-f_{1}}\right]}^{-1} \end{array}$$

(6) $$\begin{array}{c} u{\left(x\right)}_{\text{small}}={\left[1+{\left(\frac{x}{f_{4}}\right)}^{f_{3}}\right]}^{-1} \end{array}$$

where $f_{1}$ and $f_{3}$ are the spread, and $f_{2}$ and $f_{4}$ are the midpoint. The respective graphs for all used fuzzy membership functions are exemplarily provided in ESI chapter S2.

In this work, for each suitability criterion defined in Table 1, a different Fuzzy function is utilized. Recommended scaling parameters are summarized in Table 4. Since the biomass potential energy density used in this study shows loose distribution of the values within very wide range, the Fuzzy linear function is chosen to represent its membership. Instead of using the absolute minimum and maximum values, which could be heavily influenced by outliers, a suitable approach is to statistically evaluate the data distribution. The grid cells should be arranged in ascending order to derive the lower and upper quartiles, which can then be used for the minimum and maximum values for the linearly increasing function. A similar Fuzzy linear function can also be created for the wind, PV, and combined PV and wind potential energy density maps using the lower and upper quartile of the GIS datasets as the minimal and maximal values.

**Table 4:** Fuzzy membership function and recommended parameters for each criterion.

Criteria	Fuzzy function u(x)	Unit	Midpoint	Spread
Requisite				
Biomass potential	Linear increasing*	GJ_th_/km^2^		
PV potential	Linear increasing*	GWh_el_/km^2^		
Wind potential	Linear increasing*	GWh_el_/km^2^		
Combined PV/Wind	Linear increasing*	GWh_el_/km^2^		
Terrain slope	Small	Degree	$f_{4}=3000^{\circ }$	$f_{3}=5$
Water source proximity	Large and small	m	$f_{2}=1000\;m\;f_{4}=6000\;m$	$f_{1,3}=10$
Infrastructure				
Road proximity	Small	m	$f_{4}=3000\;m$	$f_{3}=10$
Railway proximity	Small	m	$f_{4}=3000\;m$	$f_{3}=10$
Oil/gas pipeline proximity	Small	m	$f_{4}=6000\;m$	$f_{3}=10$
Transmission proximity	Small	m	$f_{4}=6000\;m$	$f_{3}=10$
Refinery proximity	Small	m	$f_{4}=100,000\;m$	$f_{3}=10$
Hydrogen storage proximity	Small	m	$f_{4}=100,000\;m$	$f_{3}=10$
Airport proximity	Large and small	m	$f_{2}=1000\;m\;f_{4}=100,000\;m$	$f_{1,3}=10$
Port proximity	Large and small	m	$f_{2}=1000\;m\;f_{4}=100,000\;m$	$f_{1,3}=10$
Residential proximity	Large and small	m	$f_{2}=5000\;m\;f_{4}=100,000\;m$	$f_{1,3}=10$
Excluded areas proximity	binary	m	min=1000m	

⁎ lower and upper quartile of the GIS datasets recommended as the minimal and maximal values.

At a resolution of 1×1km, Pfennig et al. [7] excluded all areas with a slope higher than 5°. This approach is agile and convenient, but might also be arbitrary, since a large amount of forestry residues exist in the mountain area. In this current study, a Fuzzy small function with 5° as the midpoint and a spread of 5 is selected for slope, to smooth out the subjectivity.

The water source is a necessity especially for PtX and PBtX plant setups that incorporated renewable power to produce green hydrogen via water electrolysis. At the same time, small proximity to water bodies bears the high risk of contamination of the water source. Regarding these contrary conditions, a Fuzzy large and small function for water source proximity is proposed, avoiding plant locations below 1 km next to water sources as well as those further away than 6 km. However, for offshore wind turbines, since the distance to water source is always 0, the Fuzzy membership value for this criterion is always 1.

In the previous study by Jayarathna et al., the Fuzzy function for infrastructure proximity is given as Fuzzy large and small function, considering the restrictions on the proximity of construction activities based on the Euclidean distance from the centerline of the road (100 m) [6]. However, in the present study, since the cell size for each pixel (1000 m) already exceeds the restriction, only Fuzzy small function would be applied to road, railway, oil pipeline and electricity transmission line proximity. The midpoint for road and railway proximity is set at 3000 m, while the midpoint for oil pipeline and electricity transmission line proximity is set at 6000 m. For liquid fuel production, ports can also play an important role in product distribution. Here, a proximity within 100 km is considered beneficial and a minimum distance of 1 km is proposed to avoid any disturbance. Also, a nearby refinery could have a positive influence considering the possibility of direct utilization or retrofitting. In this regard, a Fuzzy small function with a midpoint of 100 km is applied. If, for example SAF is the main product of any BtX, PtX or e-/PBtX process, airports can also be included as potential demand side criteria. Similar to port proximity, a maximum distance of 100 km is considered suitable while a minimum distance of 1 km must be granted.

Proximity to residential area indicates areas with sufficient population to provide adequate work force (see Section 1). A Fuzzy small function with a midpoint of 100 km is used to include areas within 100 km of all urban and suburban residential areas, and a Fuzzy large function with a midpoint of 5 km is used to avoid any disturbance.

### Weighted overlay and suitability mapping

After processing the GIS data for selected criteria using pairwise comparison and fuzzy normalization, the next step in the process is typically a weighted overlay. In general, weighted overlay is a GIS-based technique used to combine multiple raster layers representing different criteria into a single composite raster layer. The resulting map shows the overall suitability of different locations based on the selected criteria. In a final step, the exclusion map from exclusion analysis is combined with the weighted overall of suitability criteria layers, yielding the actual suitability map to identify the most suitable locations for the BtX, PtX or e-/PBtX plants.

**Fig. 6:**
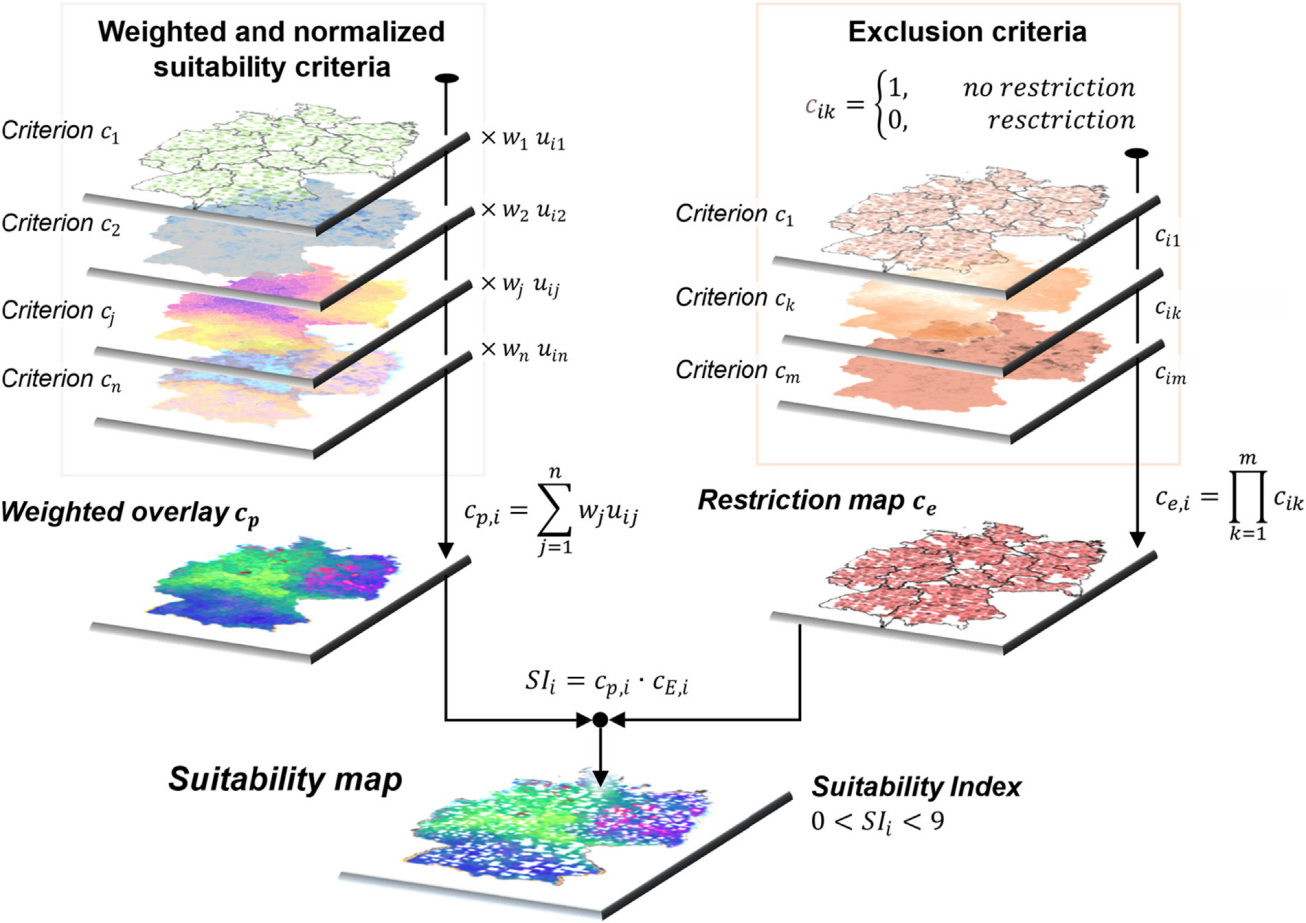
General Methodology for weighted overlay of GIS suitability criteria, exclusion analysis using exclusion criteria and final suitability mapping.

To generate a unified suitability map, each cell i in each raster corresponding to a criterion j is multiplied by its respective criteria weight $w_{j}$ and fuzzy membership function $u_{ij}$ as shown in Fig. 6. Conflicting land use areas that are specific to wind and PV potential sites are also excluded during this step using the same methodology as described below. For wind potential, land use types that include all kinds of forestry are excluded. While for PV potential, both crop lands and forestry are regarded as unsuitable. The resulting n layers are then combined through a weighted overlay with $c_{p,i}$ as the final value of the *i*^th^ grid of the weighted overlay.

For exclusion analysis, a raster layer is created for each of the m exclusion criteria from Table 2. All restricted areas which are considered unsuitable for plant siting are to be excluded from the weighted overlay. A buffer zone of 1 km is set up for each criterion to avoid the restricted area. For each cell i of each layer k, a $c_{ik}$ value of 1 is assigned to all areas without restriction, and a value of 0 to all restricted areas. A combined layer is generated from the multiplication of all layers yielding $c_{e,i}$ as the final value of the *i*^th^ cell of the restriction map shown in Fig. 6.

Using the weighted overlay from FAHP in superimposition with the restriction map from exclusion analysis, a single raster layer is constructed as the land suitability map for construction of fuel production plants. This holistic map contains the information regarding both the preference, which includes requisite, infrastructure, and environmental factors, as well as imposed restrictions. For each grid, a Suitability Index (SI) is proposed as a numerical assessment of how well each grid is suited for the placement of fuel production facilities. The SIs calculated for each cell in the suitability map are categorized into 10 groups, ranging from 0 to 9. A rating of 0 designates an entirely unavailable area, while a higher SI indicates a greater suitability for the specified criteria [61].

## Conclusion

Identifying suitable locations for sustainable fuel production is a complex decision-making process that requires the integration of spatial data, environmental aspects, and stakeholder preferences. In the context of the presented methodology a GIS-based methodology followed by a FAHP is used to assess the suitability of these locations.

The methodology involves selecting key criteria based on a comprehensive discussion of BtX, PtX and e-/PBtX process specific requirements. A short list of recommended suitability and exclusion criteria including feedstock and infrastructure requirements as well as environmental concerns is provided. The challenges associated with collecting, and processing spatial data using GIS in the fuel producing context are discussed, highlighting the technical complexities involved.

Applying exclusion criteria to unsuitable areas, the FAHP is used as MCDA method to determine the optimal sites for sustainable fuel production. A weighted overlay is employed as a spatial analysis technique to combine the selected criteria assessed using FAHP into a single suitability map. This process involves quantifying criteria data, which is then weighted according to its importance using pairwise comparison. Pairwise comparison matrices and the resulting weighting factors for SAF-specific BtX, PtX, and e-/PBtX processes are presented.

Suitability criteria are normalized using individually selected and parameterized Fuzzy membership functions. Exclusion analysis introduces exclusion criteria for conflicting land use resulting in a restriction map to be excluded from the weighted overlay. The so-derived suitability map can then be used to identify and prioritize potential plant locations based on their overall suitability scores ranging from 0 to 9 for each of the possible processes (BtX, PtX, e-/PBtX). Finally, the implications of the so-produced suitability maps and their limitations are discussed, emphasizing their practical applicability in decision-making for sustainable fuel production initiatives.

## Limitations

Overall, the presented methodology provides a structured and systematic approach to eliciting, quantifying, and incorporating decision-makers' preferences into the evaluation of suitable locations for sustainable fuel production. However, these suitability maps require proper interpretation to evaluate the individual locations. Furthermore, there are some common limitations of suitability mapping including general data availability and quality, subjectivity and sensitivity of selected parameters and the complexity of interpretation.

When identifying suitable locations for sustainable fuel production facilities, specifically focusing on BtX, PtX, and their combinations (eBtX, PBtX), the methodology described above is well suited to reduce the number of possible locations to a finite number of most suitable alternatives. Each location on a map is represented by its performance in multiple criteria, including feedstock availability, transport, and other infrastructure as well as environmental impact. Concentrating this performance into a single metric, the suitability index (SI), allows to prioritize the most suitable potential locations for the fuel production facilities.

To make informed decisions about where to locate these facilities, further analysis, and stakeholder consultations, are recommended. One important step is a more detailed process analysis, including techno-economic assessments (TEA), as well as environmental impact or lifecycle assessments (LCA). While a TEA can be used to quantify the expected costs associated with setting up and operating the facilities, as well as the expected benefits in terms of fuel production and potential revenue, the LCA evaluates the potential environmental effects of the facilities at each location in more detail. To conduct these analyses, the most appropriate technology for the fuel production facilities must be selected. Based on the suitability map and process development tools factors such as feedstock availability, infrastructure requirements, and environmental impact can be combined with BtX, PtX and e-/PBtX specific technologies. The location of the plant also determines the size of the production facility and at the same time defines the transportation distance, e.g. for the supply of biomass or water or the distribution of products. This results in a conflict of interest between having a large production facility for economies of scale and minimizing transportation distances to reduce costs and environmental impact. A detailed TEA including transportation costs, capital expenditure, and operational costs at different scales can help to determine the optimal facility size that balances these factors. Additionally, GIS-based network analysis can be used to evaluate different transportation methods or routes to minimize distances or to compare alternative plant locations.

While such detailed process analysis is time consuming, focusing on the most suitable locations, i.e. those with the highest SI, allows to focus on the essential locations. Also, location pre-selection allows for site specific boundary conditions to be incorporated into the analysis. Depending on feedstock characteristic and availability, for example, different technologies and processes are to be compared. It is also crucial to consider local regulations pertaining to environmentally, geographically, and culturally significant protected natural and man-made areas. Thus, since suitability mapping only provides information about the physical suitability of land for different uses but does not consider other important factors such as economic feasibility, social acceptability, or legal constraints, engaging with relevant stakeholders, including local communities, government agencies, and environmental organizations, is necessary. To gather feedback and address concerns related to the proposed facilities is crucial to ensure that the facilities are socially acceptable and address local needs and priorities. While the present work aims at providing a general methodology applicable to most BtX, PtX, and e-/PBtX suitability analyses, it is worth noticing that selected parameters might not be universal. Engaging with experts in the field to validate the selected criteria, weights, and methodologies can also provide valuable insights and help identify potential biases or oversights in the decision-making process. As this is an interactive process, results from process analysis, local assessment and stakeholder feedback may lead to a change in suitability analysis parameters and thus a change in its results, i.e. the mapped most suitable locations.

The presented methodology comprises a large number of input parameters. Some of them are predefined and cannot be changed, such as the general GIS data input. The accuracy and reliability of suitability maps depend on the availability and quality of that input data. If the data used to create the maps are outdated, incomplete, or inaccurate, it can lead to errors in the suitability assessment. In addition, the scale and resolution of the suitability mapping is mainly determined by the available input data. Even if detailed data is available, fine-scale mapping may be resource-intensive and costly, while coarse-scale mapping may lack the detail needed for specific decisions.

Yet, most assumptions and parameters are user-defined and must be carefully selected. These primarily include the decision criteria that are included in the suitability and exclusion analysis. The weighting in the pairwise comparison and the fuzzy membership functions used also play a crucial role in the decision-making process and have a massive influence on the result. The process of defining criteria, weighting them, and interpreting the results can be subjective and influenced by the biases of the analysts or decision-makers involved. Though the systematic approach including pairwise comparison, consistency check and expert consultation, subjectivity can affect the reliability and credibility of the resulting suitability maps. To handle uncertainty and limitations in decision-making for sustainable fuel production facilities, especially regarding BtX, PtX, and e-/PBtX processes, several strategies can be employed. Sensitivity analysis can be used to evaluate the impact of changes in input parameters, criteria weights, or fuzzy membership functions on the final suitability map. Existing GIS software tools allow the automation of such analysis to better understand the robustness of the model and the sensitivity of the results to different assumptions. Furthermore, different scenarios might be considered taking into account changing boundary conditions. Future conditions, e.g., climate change, changes in biomass availability, technological advancements, etc., may have a bigger influence on site selection than today's. To assess how these scenarios would affect the suitability of locations can provide insights into long-term sustainability and adaptability of the chosen locations.

Furthermore, the suitability maps derived using the presented methodology are limited in the context of land resource allocation. GIS data on biomass residues typically does not account for seasonal changes, depending on the specific dataset and its purpose. Most GIS datasets provide static information on biomass residues, representing a yearly average of biomass availability. However seasonal variations in biomass data could be incorporate by using, for example, satellite-based remote sensing data monitoring vegetation growth and biomass accumulation over time, allowing for the creation of time-series datasets that capture seasonal changes in biomass residues. Additionally, field surveys and biomass monitoring programs typically do collect data at regular intervals to track seasonal variations in biomass availability. Biomass growth models validated using that data might be a suitable option too. Since for the general suitability analysis proposed in this work, the inclusion of seasonal changes in GIS data on biomass residues is not required, the quantities averaged over the year are sufficiently detailed.

While CES-SAFAHP provides information about the suitability of different land parcels for the use as BtX, PtX, or e-/PBtX plant location, this information alone cannot determine how land should be allocated among conflicting different uses. To make decisions about land allocation, not only the suitability of the land but also the demand for the different land uses must be considered. In other words, it's not enough to know which land is suitable for BtX, for example; you also need to know how much demand there is for BtX compared to other uses like urban development or conservation. While this conflict is relatively minor in terms of the area actually occupied by the (e-/P)BtX facility, the allocated feedstock are almost always part of a competing market. It is therefore too simplistic to claim that the suitability map alone can determine the best distribution of land uses. However, to make informed decisions about land use allocation, one must also consider economic factors, such as market demand for different land uses, as well as social and environmental factors. Thus, a more integrated approach might be required to combine land suitability analysis with other land use modeling and economic analysis to ensure that land is allocated in a way that meets the needs of society while also preserving the environment [62].

## Declaration of generative AI and AI-assisted technologies in the writing process

During the preparation of this work the authors used Open AI's ChatGPT to increase language. After using this tool/service, the authors reviewed and edited the content as needed and take full responsibility for the content of the publication.

## CRediT authorship contribution statement

**Marcel Dossow:** Project administration, Conceptualization, Methodology, Writing – original draft, Visualization. **Mengxi Chen:** Investigation, Software, Methodology, Writing – original draft. **Hartmut Spliethoff:** Resources, Funding acquisition. **Sebastian Fendt:** Supervision, Writing – review & editing.

## Declaration of competing interest

The authors declare that they have no known competing financial interests or personal relationships that could have appeared to influence the work reported in this paper.

## Data Availability

No data was used for the research described in the article.

## Appendix Supplementary materials

Image, application 1
