# Supplementary material for: Advancing GIS-based suitability analysis of BtX, PtX, PBtX, and eBtX facilities using the fuzzy analytic hierarchy process
Source: MethodsX. 2025 Jan 29;14:103194. doi: 10.1016/j.mex.2025.103194 (PMC11847470; doi:10.1016/j.mex.2025.103194)
Supplement: Supplementary file 1 [file mmc1.pdf]

Chair of Energy Systems
School of Engineering and Design
Technical University of Munich

# Electronic Supplementary Information (ESI)

Advancing GIS-based suitability analysis of BtX, PtX, PBtX, and eBtX facilities using the fuzzy analytic hierarchy process

**Marcel Dossow, Mengxi Chen, Hartmut Spliethoff, and Sebastian Fendt**

Chair of Energy Systems, School of Engineering and Design, Technical University of Munich

marcel.dossow@tum.de
sebastian.fendt@tum.de

October, 2024

### ESI submitted with MethodsX paper:

| | |
|---|---|
| **Name of your method** | TUM Chair of Energy Systems GIS-based Suitability Analysis using the Fuzzy Analytic Hierarchy Process for optimal BtX, PtX, PBtX, and eBtX plant siting (CES-GIS-SAFAHP) |
| **Subject area** | Energy & Suitability analysis, mapping, and site selection to produce sustainable fuels and chemicals from biomass and electricity |

# S1 CES-GIS-SAFAHP Workflow Algorithms

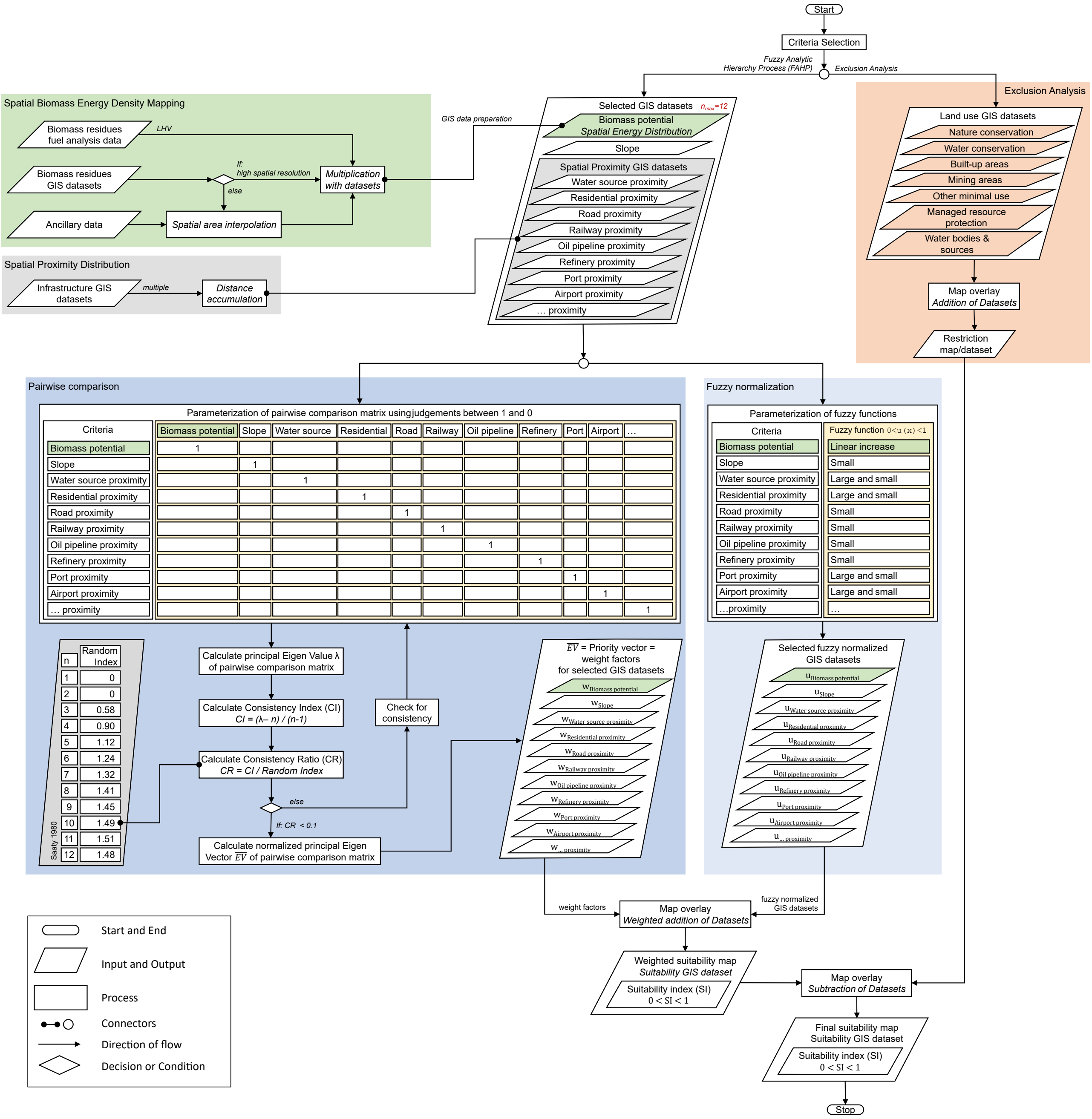


**Figure S1** Simplified workflow algorithm applying the TUM CES-GIS-SAFAHP method for optimal BtX plant siting as introduced in the MethodsX paper

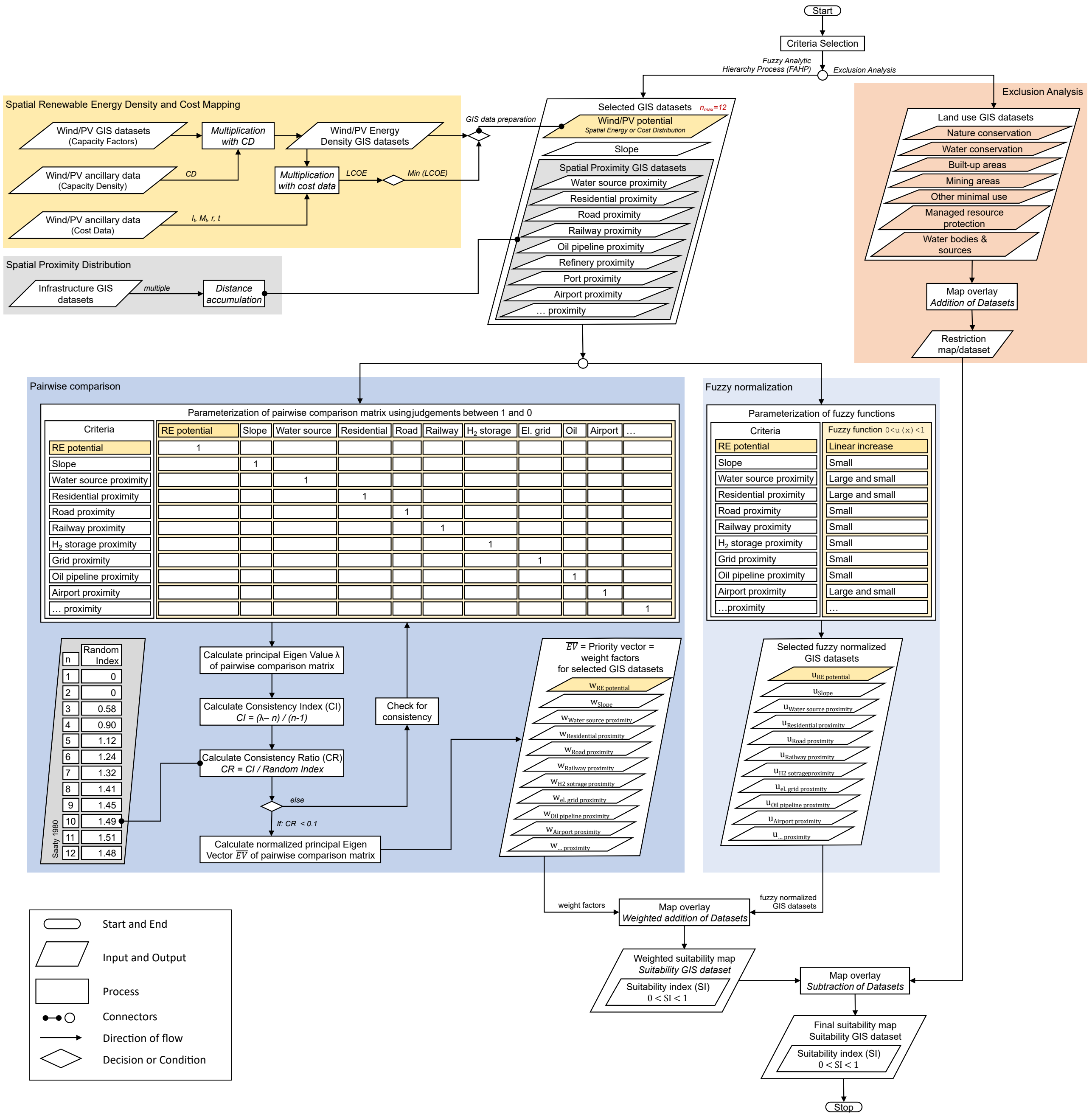


**Figure S2** Simplified workflow algorithm applying the TUM CES-GIS-SAFAHP method for optimal PtX plant siting as introduced in the MethodsX paper

# S2 Fuzzy Normalization

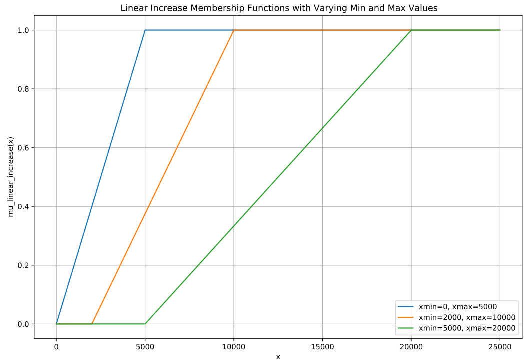


**(a)** linear increasing

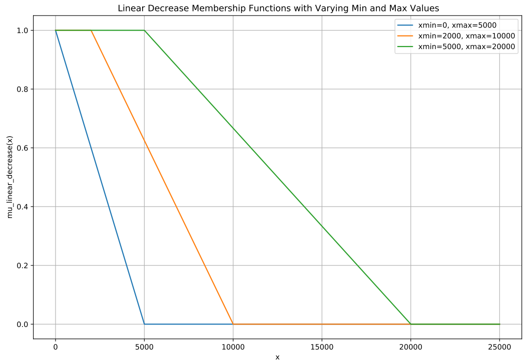


**(b)** linear decreasing

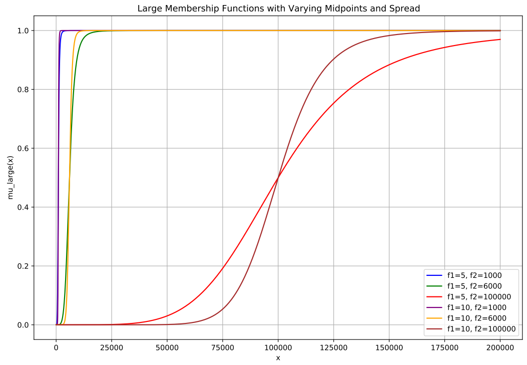


**(c)** large)

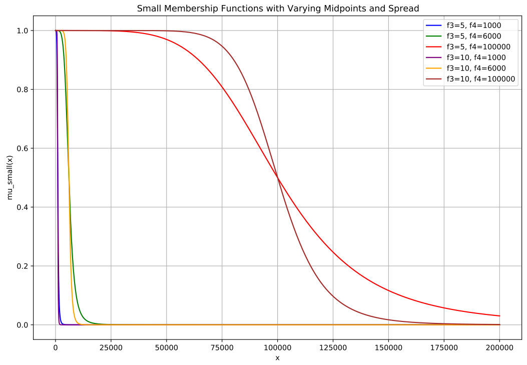


**(d)** small)

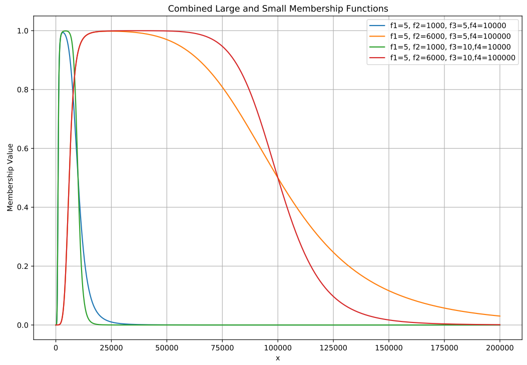


**(e)** large and small)

**Figure S3** Representation of employed Fuzzy membership functions with varying parameters
